# Supplementary material for: Increased in vivo transduction of AAV-9 cargo in Alport podocytes
Source: Wellcome Open Res. 2025 Jul 30;10:386. [Version 1] doi: 10.12688/wellcomeopenres.24212.1 (PMC12699215; doi:10.12688/wellcomeopenres.24212.1)
Supplement: Peer review history

# Reviewer report: Reviewer response for version 1

**DOI:** 10.21956/wellcomeopenres.26707.r139466

**Published:** 2025-12-11

**Authors:** Ludovico Graziani

**Affiliations:** 1 University of Rome “Tor Vergata”, Rome, Italy

Thank you for the opportunity to review this interesting manuscript examining AAV9-mediated transduction in an Alport (Col4a5 knockout) mouse model. The study addresses an important translational question for gene therapy in COL4-related disease. The work is promising, but several key issues require clarification, additional quantification, or textual revision before the conclusions can be supported. My main comments follow.

**1. **Your data convincingly show *enhanced* podocyte transduction in the Alport setting, but do not establish intrinsic AAV9 tropism for podocytes. Increased GFP signal may reflect disease-related permeability and GBM alterations. I recommend tempering the language throughout (Abstract, Results, Discussion) and framing the findings as “increased podocyte transduction in Alport mice,” while acknowledging alternative mechanisms.

**2. **Given known AAV9 activity in tubular epithelium, please include staining for a tubular marker (e.g., LTL or AQP1) to quantify tubular transduction. A summary table of GFP+ proportions across renal compartments (podocyte, mesangial, endothelial, tubular) would greatly clarify the distribution.

**3. **The suggestion that an intact Bowman’s capsule limits transduction in vivo is speculative. If this observation derives solely from ex vivo glomerular preparations, please clarify this explicitly and avoid attributing mechanistic in vivo significance without supporting data.

**4. **Please clarify sample sizes (animals and glomeruli per animal) for each analysis, and ensure that cell-counting and image quantification were performed blinded to genotype and treatment. If not, I recommend re-analysis under blinded conditions.

**5. **Because glomerular isolation and dissociation methods differ between experiments, please justify the approach and include representative gating plots with controls.

**6. **Recent literature indicates that multiple kidney cysts can occur in patients with COL4A3–5 variants and are increasingly recognised as part of the Alport/COL4 phenotypic spectrum (PMID: 38790225, 35155874, 38514012). It would strengthen the Discussion to briefly acknowledge this emerging phenotype and comment on whether the Col4a5 knockout mice used here show any tubular or cystic abnormalities at the studied ages. If histological images already exist, a brief check for cysts and a short statement about their presence or absence would be valuable, especially since tubular architecture can influence renal access of AAV vectors.

 The study is well executed and highly relevant, but important quantitative and interpretive weaknesses remain. I recommend **major revision** with the additions, clarifications, and moderations above. Addressing these points will considerably enhance the manuscript’s robustness and translational impact.

Is the work clearly and accurately presented and does it cite the current literature?

Yes

If applicable, is the statistical analysis and its interpretation appropriate?

Yes

Are all the source data underlying the results available to ensure full reproducibility?

Yes

Is the study design appropriate and is the work technically sound?

Yes

Are the conclusions drawn adequately supported by the results?

Partly

Are sufficient details of methods and analysis provided to allow replication by others?

Yes

Reviewer Expertise:

Medical Genetics

I confirm that I have read this submission and believe that I have an appropriate level of expertise to state that I do not consider it to be of an acceptable scientific standard, for reasons outlined above.

# Reviewer report: Reviewer response for version 1

**DOI:** 10.21956/wellcomeopenres.26707.r139469

**Published:** 2025-12-11

**Authors:** Hassan Saei

**Affiliations:** 1 Laboratory of Hereditary Kidney Diseases, Institut Imagine Institut des Maladies Genetiques, Paris, Île-de-France, France

This is a well-executed study with good quality data and the manuscript is clearly written. Similar approches have been reported AAV9 tropism to podocytes in the X-linked Alport syndrome mouse model (Meei-Hua Lin et al., Kidney International 2025). Nevertheless, several changes would strength the scope and relevance of the manuscript. 

 Main commenst:

 1. Could the authors clearly distinguish AAV9 tropism to podocytes in normal vs abnormal GBM (and intact vs injured GFB) from therapeutic application in Alport syndrome?

 The discussion appropriately acknowledges in the limitations that AAV9 is not an ideal delivery vehicle for COL4A5 replacement, given that the gene’s coding sequence exceeds the accepted maximum packaging capacity of AAV. However, the manuscript frequently states- from the Background onward- that “AAV is a promising approach for treating Alport syndrome”. This claim could be valid if the authors explicitly discuss a bypass strategy, such as a dual AAV trans-splicing/trans-gene reconstitution approach, as a realistic route to overcome vector capacity constraints.

 2. Did the authors evaluate the expression of known AAV9 entry receptor (like AAVR) on podocytes (immortalized human, isolated primary mouse podocytes, and in vivo mouse podocytes), as well as mesangial and parietal epithelial cells within their glomerular isolations? This could explain whether the observed differences in transduction, particularly the higher signal in mesangial cells and glomerulus-adjacent compartments during isolation are driven by cell accessibility due to GBM/Bowman’s capsule disruption, or by relative receptor abundance in non-podocyte populations present in the current glomerular preparations.

 3. To interpret the “increased transduction in Alport podocytes,” it is important to highlight vector access (GBM disruption leading to more particles reaching podocytes) from changes in the podocyte itself (e.g., higher entry-receptor levels, as mentioned in point 2), or both.

 It is important to highlight in the discussion the difference between isolated/immortalized podocytes (they lose the expression of key genes) and in vivo podocytes. 

 4. How the authors discuss higher transduction compared to loss of therapeutic potential by loss of podocytes?

 5. Could authors discuss a bit more, how generalizable their findings are across COL4A5 genotypes? KO compared to missense variants with missfolded protein network?

 6. AAV9 is known to transduce other renal cell types, including tubular epithelial cells. It is important to briefly report the off-target patterns observed in the current in vivo models across MOIs.

 7. The authors studied an X-linked Alport mouse model but did not include direct GBM staining. In this model, correlation between GBM thickening and transduction of adjacent cells could be assessed, and examined in female XLAS mice with segmental collagen IV[α5] deposition.

Is the work clearly and accurately presented and does it cite the current literature?

Yes

If applicable, is the statistical analysis and its interpretation appropriate?

I cannot comment. A qualified statistician is required.

Are all the source data underlying the results available to ensure full reproducibility?

Yes

Is the study design appropriate and is the work technically sound?

Partly

Are the conclusions drawn adequately supported by the results?

Partly

Are sufficient details of methods and analysis provided to allow replication by others?

Partly

Reviewer Expertise:

Human genetics, Nephrogenetics, Bioinformatics

I confirm that I have read this submission and believe that I have an appropriate level of expertise to confirm that it is of an acceptable scientific standard, however I have significant reservations, as outlined above.

# Reviewer report: Reviewer response for version 1

**DOI:** 10.21956/wellcomeopenres.26707.r139468

**Published:** 2025-11-25

**Authors:** Emine Bilge Caparali

**Affiliations:** 1 Internal Medicine PSTP Resident at UT Southwestern, Dallas, USA

This study helps address a major challenge in Alport syndrome gene therapy: achieving efficient delivery of therapeutic molecules into kidney. The authors present a clear and well-written manuscript with straightforward results. However, there are several minor points in the Methods and Discussion sections that I believe should be addressed/clarified to strengthen the work.

 Methods: 

 1- In the *“Glomerular isolation and cell culture”* subsection, it should be clarified that a portion of the isolated glomeruli was used to generate primary podocyte cultures, while another portion was used directly for AAV9 transduction experiments. Additionally, the authors should specify the number and age of mice used in each group (WT and Alport) for the glomerular isolate–derived podocyte cultures to ensure clarity and reproducibility.

 2- For the ex-vivo glomeruli experiment: authors should clarify how many 8-week old mice were used in each group.

 3- "At the defined study endpoint, four weeks post-injection, mice were humanely culled, and tissues (kidneys and liver) were collected."

  - The authors should clarify the rationale for selecting a four-week endpoint after AAV9 transduction.

 4- For the flow cytometry experiments, the authors should provide a rationale for using magnetic isolation with Dynabeads rather than mechanical isolation that was used in transduction experiment.

 Discussion: 

 1- A brief explanation should be added to clarify why the presence of an intact Bowman’s capsule would affect transduction efficiency, as noted in the Results section: *‘Overall, the ex vivo assay allows comparison of AAV9-GFP transduction efficiency in glomeruli and demonstrates that an intact Bowman’s capsule limits the transduction of glomerular cells.’*”

 Structurally, the presence of Bowman’s capsule should not limit AAV9-GFP delivery, as the vector was administered via peripheral veins and would reach glomerular cells through the glomerular capillaries. Moreover, if an intact Bowman’s capsule truly restricts glomerular transduction, this would raise concerns about the suitability of the autosomal Alport syndrome model, in which Bowman’s capsule remains intact.

Is the work clearly and accurately presented and does it cite the current literature?

Yes

If applicable, is the statistical analysis and its interpretation appropriate?

I cannot comment. A qualified statistician is required.

Are all the source data underlying the results available to ensure full reproducibility?

Yes

Is the study design appropriate and is the work technically sound?

Partly

Are the conclusions drawn adequately supported by the results?

Yes

Are sufficient details of methods and analysis provided to allow replication by others?

Partly

Reviewer Expertise:

Nephrology

I confirm that I have read this submission and believe that I have an appropriate level of expertise to confirm that it is of an acceptable scientific standard.

# Reviewer report: Reviewer response for version 1

**DOI:** 10.21956/wellcomeopenres.26707.r134220

**Published:** 2025-10-07

**Authors:** Cheng Xue

**Affiliations:** 1 nephrology, shanghai changzheng hospital, Naval Medical University (Second Military Medical University), Shanghai, China

This study addresses an important technical barrier in Alport syndrome gene therapy — achieving efficient delivery of therapeutic vectors to podocytes. The authors employ an AAV9-GFP reporter to evaluate transduction efficiency in cultured podocytes, isolated glomeruli, and an in vivo Col4a5 knockout mouse model. The work is well written, the experimental procedures are detailed, and the findings provide potentially useful insights for kidney gene therapy.

 However, the central claim that AAV9 *targets* podocytes in vivo is not fully supported by the data. While GFP expression in WT1-positive cells is shown, the evidence for podocyte-specific transduction versus passive uptake due to glomerular basement membrane (GBM) disruption remains insufficient. Additional quantitative and spatial validation would be needed to substantiate the conclusion of selective podocyte tropism.

 Major Comments

1. Specificity of AAV9 for Podocytes

The manuscript repeatedly refers to “podocyte targeting,” but the presented data primarily demonstrate *enhanced* podocyte transduction in Alport mice, not *specific* tropism.

 Because AAV9 is known to transduce multiple renal cell types (e.g., mesangial, interstitial, tubular, hepatic cells), the higher GFP signal in Alport podocytes may result from increased permeability of the glomerular filtration barrier rather than intrinsic podocyte selectivity.

 The authors should temper the wording throughout the text (including the Abstract and Discussion) to emphasize enhanced podocyte transduction in Alport pathology, rather than “AAV9 targeting podocytes.”

 2 Evidence for Colocalization: The conclusion that AAV9-GFP transduces podocytes relies mainly on WT1/GFP immunofluorescence overlap. However, the manuscript does not provide quantitative metrics (e.g., Pearson or Manders correlation coefficients) or representative single-z-plane images confirming true colocalization.

 The provided figures show merged projections, which can exaggerate overlap. The authors should provide Z-stack or Airyscan single-plane images and quantify the degree of colocalization using standard image-analysis coefficients.

  3 Controls for Non-podocyte Transduction: The study includes PDGFRβ (mesangial) and CD31 (endothelial) markers but does not evaluate tubular or interstitial cells, which are common AAV9 targets.

 Additional staining with LTL (proximal tubule) or AQP1/AQP2 would clarify whether GFP expression outside glomeruli contributes significantly to the observed signal.

 4 Quantitative Validation of Vector Uptake: The study lacks qPCR or RT-qPCR quantification of AAV genomes or GFP mRNA in kidney tissue. Including this would strengthen claims of dose- and disease-dependent delivery efficiency.

 5 Interpretation of Disease-dependent Uptake: The increase in GFP expression at later disease stages is compelling, but the authors should discuss in greater depth whether this reflects passive leakage through the damaged GBM rather than active viral tropism.

 Minor Comments

1. The sample size (n = 3–4 mice/group) is small; the exploratory nature of the study should be explicitly stated in the Discussion and Limitations sections.
2. The authors may wish to add 3D renderings or surface plots to illustrate the localization of GFP within the glomerular tuft.
3. Figure legends should clearly indicate the imaging modality (confocal vs. slide scanner) and whether images are single optical sections or projections.
4. The term “targeting” should be replaced with more precise wording such as “enhanced transduction” or “preferential delivery under pathological conditions.”
5. Please clarify whether the quantification of GFP+/WT1+ cells was performed blinded to genotype and treatment group.

Is the work clearly and accurately presented and does it cite the current literature?

Yes

If applicable, is the statistical analysis and its interpretation appropriate?

Yes

Are all the source data underlying the results available to ensure full reproducibility?

Partly

Is the study design appropriate and is the work technically sound?

Partly

Are the conclusions drawn adequately supported by the results?

Partly

Are sufficient details of methods and analysis provided to allow replication by others?

Partly

Reviewer Expertise:

nephrology

I confirm that I have read this submission and believe that I have an appropriate level of expertise to confirm that it is of an acceptable scientific standard, however I have significant reservations, as outlined above.
